# Three segment ligation of a 104 kDa multi-domain protein by SrtA and OaAEP1

**DOI:** 10.1007/s10858-022-00409-w

**Published:** 2022-12-21

**Authors:** Stephan B. Azatian, Marella D. Canny, Michael P. Latham

**Affiliations:** Department of Biochemistry, Molecular Biology, and Biophysics, University of Minnesota, Minneapolis, MN 55455 USA

**Keywords:** Segmental labeling, Protein ligation, Side-chain methyl group labeling, Sortase A, Asparaginyl endopeptidase

## Abstract

NMR spectroscopy is an excellent tool for studying protein structure and dynamics which provides a deeper understanding of biological function. As the size of the biomolecule of interest increases, it can become advantageous to dilute the number of observed signals in the NMR spectrum to decrease spectral overlap and increase resolution. One way to limit the number of resonances in the NMR data is by selectively labeling a smaller domain within the larger macromolecule, a process called segmental isotopic labeling. Many examples of segmental isotopic labeling have been described where two segments of a protein are ligated together by chemical or enzymatic means, but there are far fewer descriptions of a three or more segment ligation reaction. Herein, we describe an enzymatic segmental labeling scheme that combines the widely used Sortase A and more recently described OaAEP1 for a two site ligation strategy. In preparation to study proposed long-range allostery in the 104 kDa DNA damage repair protein Rad50, we ligated side-chain methyl group labeled Zn Hook domain between two long segments of otherwise unlabeled *P.furiosus* Rad50. Enzymatic activity data demonstrated that the scars resulting from the ligation reactions did not affect Rad50 function within the Mre11-Rad50 DNA double strand break repair complex. Finally, methyl-based NMR spectroscopy confirmed the formation of the full-length ligated protein. Our strategy highlights the strengths of OaAEP1 for segmental labeling, namely faster reaction times and a smaller recognition sequence, and provides a straightforward template for using these two enzymes in multisite segmental labeling reactions.

## Introduction

NMR spectroscopy is an excellent tool for probing the interplay between biomolecular structure, dynamics, and function. ^15^N–^1^H backbone correlation-based experiments have provided much information in these realms, although characterization of systems > 30 kDa in molecular weight can be difficult because of decreased signal-to-noise, due to increased transverse relaxation rates, and increased spectral crowding from more, broader peaks. Fortunately, advances in deuterium incorporation, side-chain methyl labeling, and TROSY methods have unlocked the ability to observe larger systems up to 1 MDa in molecular mass (Schütz and Sprangers 2020; Alderson and Kay 2021). Although structural studies of large protein systems have historically focused on one domain or subunit at a time (i.e., a so-called “divide-and-conquer” approach), it has become clear in recent years that a complete understanding of biological systems often requires the presence of proteins in their native multi-domain or multi-subunit forms. Isotopic labeling strategies limit the NMR signals to certain functional groups (e.g., side-chain methyl or aromatic groups); however, standard heterologous expression systems make further limiting the incorporation of NMR active nuclei to a single domain within a full-length protein impossible. Nevertheless, labeling of an individual domain within a multi-domain protein can be achieved through the use of segmental isotopic labeling strategies. These strategies use various protein ligation approaches to form a complete protein sequence from separately expressed unlabeled and labeled peptide segments (Schmidt et al. 2017; Nuijens et al. 2019; Vogl et al. 2021). The demand for these strategies has led to the emergence of a diverse toolset of synthetic and enzymatic ligation methods (Skrisovska et al. 2010; Vogl et al. 2021). For example, in intein-mediated protein ligation (IPL), the C-terminus of a recombinantly expressed protein fused to an intein is converted to an ⍺-thioester. Subsequent transesterification and N-acyl shift reactions replace the thioester with an amide group from an N-terminal cysteine of a second polypeptide. Enzyme-mediated ligation represents an alternative approach for segmental labeling. Historically, the predominant enzyme for this technique has been a transpeptidase from *S. aureus*: Sortase A (SrtA) (Antos et al. 2016). Although active in its wild-type form, SrtA suffers from low catalytic efficiency and dependence on cofactors. A recombinantly expressed, calcium-independent, hepta-mutant of SrtA ligates C- and N-termini with respective “LPXTG” and “G” motifs at considerably higher yields than the wild type enzyme (Jeong et al. 2017). Recently, a relatively novel endopeptidase, *O. affinis* Asparaginyl Endopeptidase 1 (OaAEP1), has emerged as a more efficient alternative to SrtA with a smaller sequence recognition requirement, catalyzing peptide bond formation between polypeptides with “NXL” and “GL” sequences at the C- and N- termini, respectively (Harris et al. 2015).

**Fig. 1:**
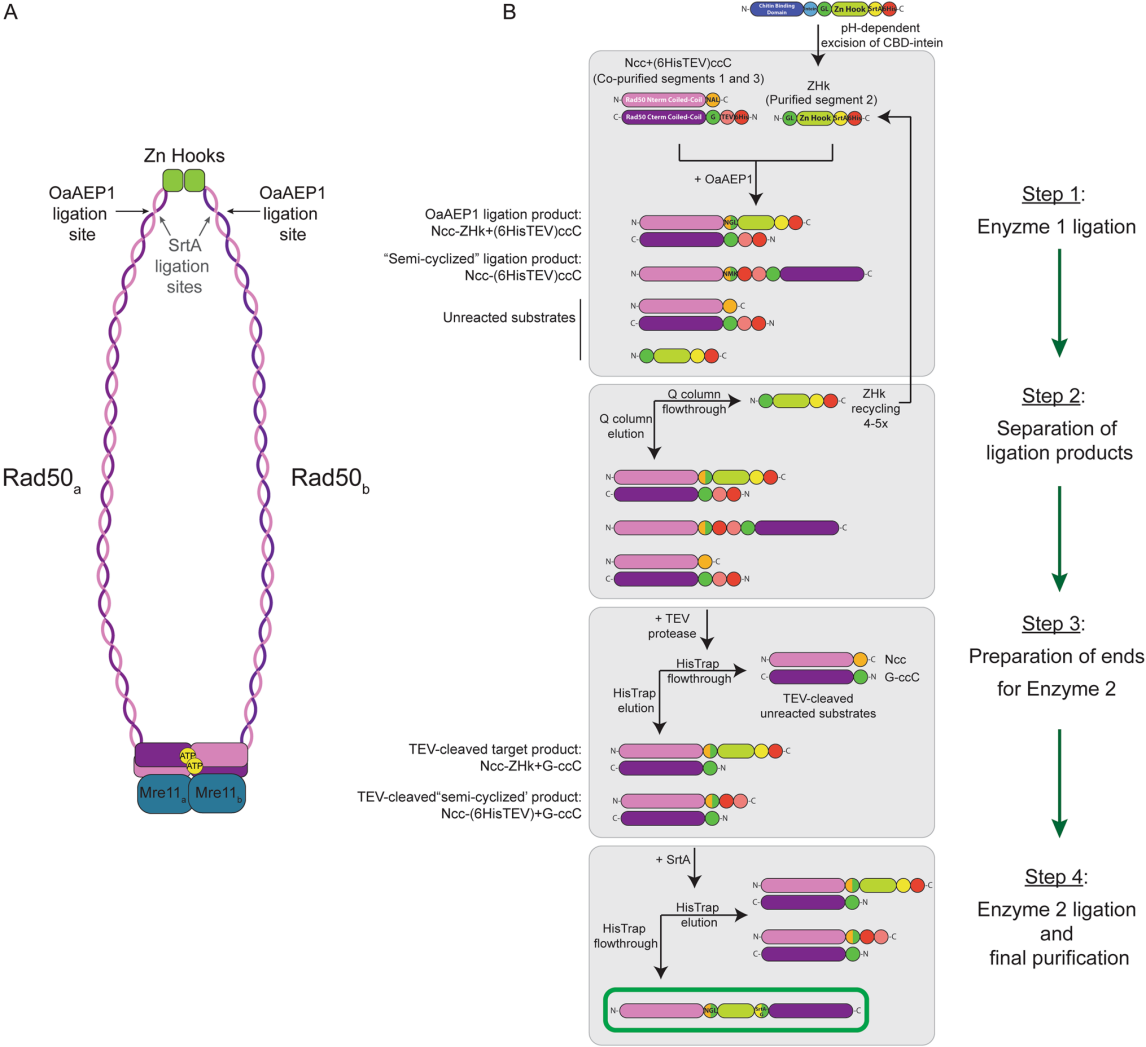
Ligation scheme for the segmental isotopic labeling of *Pf* Rad50. **A** Cartoon of the Mre11_2_-Rad50_2_ (MR) complex showing the overall architecture and the approximate locations of the two ligation sites on each Rad50 protomer. **B** Three-segment *Pf* Rad50 ligation and purification scheme. Individual domains and recognition sequences are represented as the following—light purple rectangle, Rad50 N-terminal NBD and coiled-coil domain (Ncc, segment 1; aa 1–389); orange circle, OaAEP1 ‘NAL’ recognition sequence; dark purple rectangle, Rad50 C-terminal coiled-coil domain and NBD (ccC, segment 3; aa 502–882); green circle, ‘G’; salmon circle, ‘ENLYQG’ TEV protease recognition sequence; red circle, 6His tag; blue rectangle, chitin binding domain; light blue circle, intein; green circle, OaAEP1 ‘GL’ recognition sequence; lime green rectangle, Rad50 Zn Hook domain (ZHk, segment 2; aa 390–501); and yellow circle, SrtA ‘PLTG’ recognition sequence. The desired end product (i.e., ligated full-length Pf Rad50) is outlined in the green box at the bottom of the scheme

The Mre11-Rad50 complex (MR) is essential for DNA double-strand break repair in all organisms (Paull 2018; Syed and Tainer 2018; Reginato and Cejka 2020). It has been suggested that conformational changes in the globular, enzymatic nucleotide binding domain (NBD) of the Rad50 protein propagate long-range allosteric changes along its coiled-coil domains out to the apical Zn Hook dimerization domain, a distance of 600–800 Ångstroms (Fig. 1A) (Hohl et al. 2011, 2015); however, there is no direct high-resolution characterization of these potential allosteric changes. The large size and inherent flexibility of the coiled-coils makes the characterization of the full-length MR complex by X-ray crystallography impossible, and the large number of side-chain methyl groups in a standard side-chain methyl group labeled Rad50 sample would lead to severe signal overlap in methyl-TROSY spectra. In fact, high-resolution crystal structures and NMR studies of the MR complex or Rad50 Zn hook domain have only been determined using truncated constructs (Syed and Tainer 2018; Beikzadeh and Latham 2021). Thus, the full-length complex has largely been studied by atomic force microscopy (AFM) (De Jager et al. 2001; van Noort et al. 2003) and more recently by cryo-electron microscopy (cryo-EM) (Käshammer et al. 2019; Gut et al. 2022). Methods for selective isotopic labeling of the coiled-coil and Zn Hook regions are currently unexplored but would allow for a structural and dynamic characterization of potential long-range allosteric communication within the protein by NMR spectroscopy. In this paper, we describe the combination of SrtA and OaAEP1 enzymatic strategies to ligate a three-segment construct of full-length *P. furiosus* (*Pf*) Rad50, a multi-domain protein of ~ 104 kDa (Fig. 1A), so that only the apical Zn Hook domain is isotopically side-chain methyl group labeled in an otherwise unlabeled protein. In this approach, six mutations (TPLL → PLTG for SrtA and LGD → NGL for OaAEP1) to Rad50 are introduced and are limited to the ligation sites; moreover, these mutations do not cause any deleterious effects on MR activity. 2D ^13^C,^1^H methyl-TROSY HMQC and ^1^H R_2_ data on side-chain methyl group Zn Hook-labeled *Pf* MR (300 kDa as the M_2_R_2_ tetramer) showed the expected changes in chemical shifts as compared to the isolated Zn Hook, particularly at the sites of ligation, and increased transverse relaxation rates indicating that the labeled Zn Hook segment was successfully ligated in between the N- and C-terminal segments of Rad50. Our work gives an example of using the efficient OaAEP1 enzyme for segmental labeling coupled with NMR-based studies and additionally provides a framework for future studies where multiple ligation approaches are necessary to insert NMR active nuclei into a single domain of a larger, multi-domain protein.

## Materials and methods

### Plasmids and cloning

**Fig. 2:**
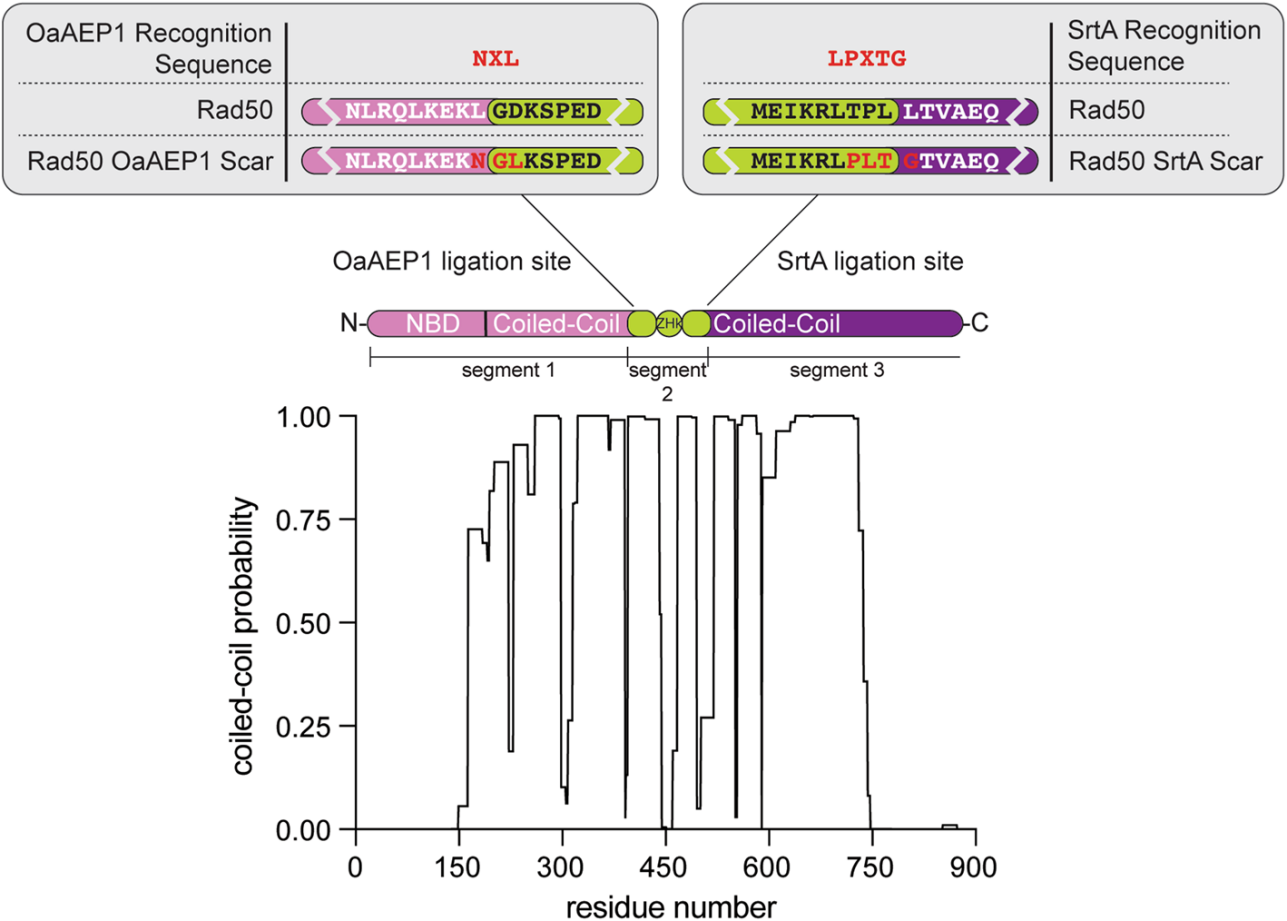
OaAEP1 ligation sites within *Pf* Rad50. Graph of NCOILS data indicating the coiled-coil propensity along the primary sequence of *Pf* Rad50. Above is a domain structure of Rad50 delineating the three segments used in the ligation scheme. The top shows the OaAEP1 and SrtA Ligation sites highlighting the primary sequences of *Pf* Rad50 before and after modification with ligation scars

The expression plasmid containing OaAEP1 (pBHRSF184) was a gift from Hideo Iwa (Addgene plasmid # 89482; http://n2t.net/addgene:89482; RRID:Addgene_89482) (Mikula et al. 2017). To facilitate faster ligation rates, the Cys at position 247 was changed to an Ala using site-directed mutagenesis (Yang et al. 2017). The Sortase A (SrtA) plasmid was graciously gifted by the Kay laboratory (Rosenzweig et al. 2015). A non-cleavable 6His nickel affinity tag was inserted after the C-terminus of the SrtA. The full-length *P. furiosis* (*Pf*) Rad50 gene was amplified from genomic DNA (ATCC) and first cloned into the pET29 expression plasmid (Novagen) between the NcoI and NotI restriction enzyme sites. The N-terminal segment 1 (aa 1–389; Fig. 1B, light purple oval) of *Pf* Rad50 was then subcloned into the pTWINI expression plasmid (New England Biolabs) between NdeI and SapI. For OaAEP1 recognition, the C-terminus of this segment was modified from “LGD” to “NAL” (Table 1, Ncc and Fig. 1B, orange circle) (Mikula et al. 2017). The C-terminal segment 3 (aa 502–882; Fig. 1B, dark purple oval) of *Pf* Rad50 was subcloned into the pCOLA-Duet expression plasmid (Sigma Aldrich) between NdeI and KpnI. For SrtA ligation, the N-terminal residue of this segment was mutated to Gly, and a 6His nickel affinity tag and TEV protease cleavage site were added to the N-terminus to aid in protein purification (Table 1, (6HisTEV)ccC and Fig. 1B, green, salmon, and red circles respectively). pTWINI and pCOLA-Duet expression plasmids were chosen to prevent competition of replication during co-expression by two otherwise similar origins of replication (Yamaguchi et al. 1982). The Rad50 Zn Hook segment 2 (aa 390–501; Fig. 1B, lime oval) was cloned restriction-free (van den Ent and Löwe 2006) from the genomic sequence into the multiple cloning site of pTWINI immediately following the modified *Ssp* DNAb Intein (Table 1, ZHk). The expressed Zn Hook protein will therefore have an N-terminal chitin binding domain affinity tag followed by a modified *Ssp* DNAb Intein, which allows peptide bond cleavage in response to low pH resulting in a N-terminal Gly at the end of the Zn Hook (Fig. 1B, dark blue oval, light blue circle, and green circle, respectively). The next residue of this segment (Asp), which corresponds to position “P2” in previously described OaAEP1 reactions, was mutated to a hydrophobic Leu (Harris et al. 2015) for OaAEP1 recognition (Fig. 2). The C-terminal “TPLL” sequence of this segment was mutated to the SrtA-compatible sequence “PLTG,” followed by a 6His nickel affinity tag (Fig. 1B, yellow and red circles, respectively, and Fig. 2).

**Table 1:** Definitions of abbreviations of ligation intermediates

Symbol	Protein construct	Size (kDa)
Ncc	*Pf* Rad50 N-terminal NBD-coiled-coil (aa 1 – 386)-NAL	46.7
6HisTEV	HHHHHH-ENLYFQ^G Ni^2+^ affinity + TEV cleavage site tag	3.0
(6HisTEV)ccC	(MK-6HisTEV) *Pf* Rad50 C-Terminal coiled-coil-NBD (aa 503 – 882)	44.5(+ 3.0)^a^
Ncc + (6HisTEV)ccC	Ncc non-covalently complexed with (6HisTEV)ccC	91.2(+ 3.0)^a^
ZHk	GL-*Pf* ZnHook (aa 390 – 497)-LPLT^G-6His	14.2
Ncc-ZHk	Ncc ligated to ZHk	60.7
Ncc-(6HisTEV)ccC	Ncc ligated to (6HisTEV)ccC (off-target OaAEP1 product)	91.0(+ 3.0)^a^
Ncc-6His	Linearized Ncc-(6HisTEV)ccC (after TEV cleavage)	49.5
Rad50	*Pf* Rad50	104.3

^a^Molecular weight corrections before and after cleavage with TEV protease

“^”Denotes sites of cleavage by TEV or SrtA

### Protein expression and purification

Expression and purification of OaAEP1 C247A was carried out as previously described with some modifications (Harris et al. 2015). Instead of T7 Shuffle, the protein was grown in ArcticExpress (DE3) competent cells (Agilent Technologies) at 30 °C in Luria Broth (LB) media containing 50 µg/mL kanamycin until reaching an OD_600_ of 2, after which, protein expression was induced with 0.3 mM IPTG and continued at 11 °C for 20 h. The cells were harvested in lysis buffer (50 mM Tris–HCl, 150 mM NaCl, 0.1% Triton X-100, 0.1 mM EDTA, pH 7) and lysed by homogenization. 25 mM imidazole was added to the cleared lysate which was then passed over a HisTrap HP column (Cytiva). Non-specifically bound proteins were washed off with a high salt buffer (25 mM HEPES, 1.5 M NaCl, 25 mM imidazole, pH 7), and OaAEP1 was eluted with buffer containing 300 mM imidazole. The pH was decreased to 4.5 using rapid dilution into an 8 × volume of activation buffer (50 mM NaOAc, 0.5 mM TCEP, 1.0 mM EDTA, pH 4). The diluted solution was incubated at room temperature overnight. After filtering out any precipitated protein, the soluble enzyme was further purified on a HiTrap SP HP column (Cytiva) in activation buffer using a 50–1000 mM NaCl elution gradient at pH 4. The components and ligation activity of the fractions were validated by SDS-PAGE analysis. Active enzyme was flash-frozen in liquid nitrogen and stored at -80 °C.

SrtA was expressed in *E. coli* BL21 Codon + competent cells grown at 37 °C in LB media containing 50 µg/mL kanamycin. Upon reaching an OD_600_ of 0.6, protein expression was induced with 0.3 mM IPTG and continued for 18 h at 25 °C. The cells were harvested in SrtA HisTrap Buffer A (50 mM Tris–HCl, 150 mM NaCl, 10 mM imidazole, 5 mM MgCl_2_, 10% (v/v) glycerol, pH 7.5) and lysed by homogenization. The cleared lysate was passed over a HisTrap HP column, and bound protein was eluted with buffer containing 300 mM imidazole. The sample was concentrated in a 10 kDa MWCO Millipore centrifugal concentrator and purified on a HighLoad 16/600 Superdex 200 pg sizing column (Cytiva) equilibrated in SrtA S200 Buffer (25 mM Tris–HCl, 150 mM NaCl, pH 7.5). Fractions containing the desired protein were concentrated, flash frozen, and stored at − 80 °C.

Unlabeled Rad50 segments 1 and 3 (Ncc + (6HisTEV)ccC, Table 1) were co-expressed in *E. coli* BL21 Codon + competent cells grown at 37 °C in LB media containing 100 µg/mL ampicillin and 50 µg/mL kanamycin. Upon reaching an OD_600_ of 0.7, protein expression was induced with 0.3 mM IPTG and continued for 18 h at 18 °C. The protein was purified using a previously described method for a truncated Rad50 construct (Boswell et al. 2018). Purification proceeded through the HisTrap HP column, at which point the imidazole was removed via dialysis into storage buffer (50 mM NaH_2_PO_4_/Na_2_HPO_4_, 200 mM NaCl, 5% glycerol, pH 8), and the sample was concentrated, flash frozen, and stored at − 80 °C.

Uniformly deuterated, Ileδ1-[^13^CH_3_], Leuδ/Valγ-[^13^CH_3_/^12^CD_3_], Metε-[^13^CH_3_] (ILVM)-labeled segment 2 (ZHk, Table 1) was expressed using previously described methods for recombinant expression in deuterated minimal media (Tugarinov et al. 2006; Azatian et al. 2019; Schütz and Sprangers 2020). After expression, the cells were lysed via homogenization in lysis buffer (50 mM NaH_2_PO_4_/Na_2_HPO_4_, 300 mM NaCl, 25 mM imidazole, 10 mM 2-mercaptoethanol, pH 8). The cleared lysate was loaded onto a HisTrap HP column, and the protein was eluted in buffer containing 300 mM imidazole. The pH of the sample was lowered to 6.9, and the chitin binding domain (CBD)-intein tag auto-processed over three days. The bulk of the excised CBD-intein tag was removed using the HisTrap HP column, and any negatively charged contaminants were then separated out as the elution on a HiTrap Q HP column (Cytiva) in wash buffer (50 mM NaH_2_PO_4_/Na_2_HPO_4_, 80 mM NaCl, 10 mM 2-mercaptoethanol, pH 8). Finally, to remove any remaining CBD-intein, the sample was passed over 3 mL of chitin resin (New England Biolabs) equilibrated in the same buffer. The unbound fraction containing ZHk was then concentrated, flash frozen, and stored at − 80 °C.

Mre11 protein expression and purification for Mre11-Rad50 complex was as previously described (Boswell et al. 2020; Rahman et al. 2020).

### Ligation Procedure

#### Step 1: primary ligation with OaAEP1 enzyme

The ligation protocol began by joining the Ncc and ZHk (segments 1 and 2) of Rad50 (Fig. 1B, Step 1). A mixture of Ncc + (6HisTEV)ccC complex, ILVM-labeled ZHk, and OaAEP1 were incubated at room temperature in OaAEP1 Activity Buffer (Table 2) for 20 min. Across different ligation cycles, the concentration of ZHk was kept at 100 µM, whereas the concentration of the Ncc + (6HisTEV)ccC complex increased between 25 and 100 µM with each progressive ligation cycle (see below). Reaction volumes varied between 1 and 10 mL. The concentration of OaAEP1 used for *trans*-ligation was determined from previous literature and fluctuated depending on the activity of the prep (Mikula et al. 2017). The reaction was quenched by a 1:8 dilution with Nickel Column Buffer A (Table 2), followed by removal of OaAEP1 on a HisTrap HP column. The bound sample was eluted using Nickel Column Buffer B (Table 2) and then dialyzed into 1 L Q Buffer A (Table 2). The total protein concentration of the sample was determined by UV absorbance at 280 nm (ε = 56,730 M^−1^ cm^−1^ for Ncc + (6HisTEV)ccC).

**Table 2:** Buffers used in the segmental ligation of methyl labeled ZHk into Rad50

OaAEP1 activity buffer	50 mM HEPES pH 7.5 150 mM NaCl 0.1 mM EDTA 0.5 mM TCEP
Nickel Column Buffer A	50 mM sodium phosphate pH 8 300 mM KCl 10 mM imidazole 10% glycerol 10 mM 2-mercaptoethanol
Nickel Column Buffer B	50 mM sodium phosphate pH 8 300 mM KCl 300 mM imidazole 10% glycerol 10 mM 2-mercaptoethanol
Q Buffer A	25 mM Tris–HCl pH 8 75 mM NaCl 10% glycerol 10 mM 2-mercaptoethanol
Q Buffer B	25 mM Tris–HCl pH 8 1 M NaCl 10% glycerol 10 mM 2-mercaptoethanol
SrtA Activity Buffer	25 mM Tris–HCl pH 7.65 150 mM NaCl 0.2 mM DTT

#### Step 2: Separation of ligation products and recovery of unreacted ZHk on the Q column

The reaction components of the OaAEP1 ligation (Step 1) were separated on a Q HP ion exchange column (Fig. 1B, Step 2). Unreacted ZHk was predominantly in the flowthrough, whereas the mixture of unreacted Ncc + (6HisTEV)ccC, ligated Ncc-ZnHk + (6HisTEV)ccC (i.e., the desired product), and off-target “semi-cyclized” ligated Ncc-(6HisTEV)ccC (see Results) complexes co-eluted within a gradient of Q Buffers A and B (Table 2). The recovered ZHk was recycled for a subsequent OaAEP1 ligation reaction by concentration in a 10 kDa MWCO Millipore centrifugal concentrator to < 1 mL. Fresh Ncc + (6HisTEV)ccC was added at the appropriate ratio and fresh OaAEP1 was added to start the new ligation reaction. This recycling allowed for the greatest extent of incorporation of the expensive ILVM-labeled segment. After 4–5 cycles, the recovery of Ncc + (6HisTEV)ccC complex after Step 2 was too low to justify another ligation cycle (see below).

#### Step 3: TEV cleavage and removal of unreacted substrates with the nickel column

Following elution from the Q column, the mixture of unreacted, ligated, and “semi-cyclized” products was treated with 0.6 mg of TEV protease for ~ 1–2 days at room temperature (Fig. 1B, Step 3). This step removes the 6His affinity tag from the unreacted Ncc + (6HisTEV)ccC and from the (6HisTEV)ccC subunit of the desired product and also cleaves the undesired “semi-cyclized” ligation product Ncc-(6HisTEV)ccC. Note, a 6His tag remains on the desired product (Ncc-ZHk) on the C-terminus of the ZHk. The products of the TEV reaction were once again separated on a HisTrap HP column, in which the unreacted products were in the flowthrough and the target and now cleaved “semi-cyclized” products were in the elution (Fig. 1B, Step 3).

#### Step 4: SrtA ligation and final nickel column purification

In the final step, segments 2 and 3 were ligated by SrtA (Fig. 1B, Step 4). The HisTrap elution from Step 3 and 200–250 nmol SrtA were dialyzed into 1 L SrtA Activity Buffer (Table 2) at 37 °C. An additional 200–250 nmol of fresh SrtA was added to the reaction (i.e., to the dialysis tube) every 2–3 days to maintain efficiency of the ligation reaction. The reducing environment was preserved by the addition of 0.2 mM DTT to the dialysis buffer every 2–3 days. Formation of the ligation product was monitored by SDS-PAGE gel analysis, and the reaction was stopped when the results showed diminishing returns (i.e., no further appearance of product over time). For Rad50, this was about 9 days. Any precipitation was filtered out of the sample before the SrtA ligation products were separated using a HisTrap HP column and Nickel Column Buffers A and B (Table 2). Having lost all remaining 6His affinity tags upon SrtA ligation of segments 2 and 3, the fully ligated product was predominantly in the flowthrough (Fig. 1B, Step 4). This product could then be used to make M_2_R_2_ complex or purified further on a sizing column.

### SDS-PAGE band analysis

In cases where the concentration of ligation products could not be directly measured, we utilized the ImageQuant (Cytiva) software to measure the absorbance of Coomassie-stained bands on SDS-PAGE gels. The data were processed by a rolling ball algorithm, and the resulting baselines were subtracted from the final intensity values. Within a gel, all SDS-PAGE samples were loaded with a constant amount of Ncc + (6HisTEV)ccC complex. The band corresponding to the size of the complex in OaAEP1 negative lanes was used as a control for calculating the mass per unit of absorbance of the dye in the gel. We assumed a 1:1 ratio for the non-covalent coordination of Ncc and (6HisTEV)ccC. To approximate the protein yield of the reaction, the absorbances of the ligated Ncc-ZHk band and the Ncc-(6HisTEV)ccC semi-cyclized product band were converted to mass (µg). To get total product formed, the mass was then corrected according to the proportion of sample loaded on the gel versus the volume of the total reaction.

### Rad50 and Mre11 activity assays

Rad50 ATP binding and MR nuclease activity assays were performed as previously described (Boswell et al. 2018, 2020; Rahman et al. 2020). Briefly, ATP binding affinity was assayed via fluorescence polarization of a BODIPY-labeled ATP (Life Technologies). MR nuclease activity was assayed on a 40-nucleotide dsDNA substrate with a 2-aminopurine incorporated as the eleventh nucleotide from the 3′-end of one strand. The signal of both assays was read on a BioTek Synergy Neo2 plate reader (Agilent).

### NMR sample preparation

Isolated ZHk NMR samples were prepared immediately after purification. The samples were buffer exchanged via repeated dilution/centrifugation in VivaSpin 10 kDa MWCO centrifugal filters into NMR buffer (25 mM HEPES, 150 mM NaCl, 1% glycerol, 1 mM EDTA, 0.9 mM ZnSO_4_, 0.5 mM TCEP, pD 7) containing 100% D_2_O and a metal-chelation buffer system for maintaining the supply of free Zn^2+^ ions (Padjasek et al. 2020). To form the MR complex, a concentrated 1.1 × equivalent of unlabeled *Pf* Mre11 was added to the ligated Rad50 sample. The mixture was concentrated to ~ 60 µM MR tetramer (i.e., M_2_R_2_) in Complex Buffer (25 mM Tris–HCl, 200 mM NaCl, 0.1 mM EDTA, 5% glycerol, 1 mM TCEP, pH 8). The mixture was heated at 62 °C for 30 min, allowed to cool, and then purified on a HighLoad 16/600 Superdex 200 pg sizing column (Cytiva) equilibrated in Complex Buffer. Fractions containing similar ratios of Mre11 and Rad50 were concentrated and exchanged into NMR buffer as described above for isolated ZHk.

### NMR data

NMR data were collected at 50 °C using an Agilent DD2 600 MHz (14.1 T) spectrometer equipped with a room temperature z-axis gradient HCN probe. All NMR data were processed with NMRPipe/NMRDraw (Delaglio et al. 1995) and analyzed with CCPN analysis (Vranken et al. 2005). Side-chain methyl group assignments of isolated ZHk were made by comparing the correlations observed in a 3D ^13^C,^13^C,^1^H HMQC-NOESY-HMQC (250 ms mixing time) to distances in the *Pf* Zn Hook crystal structure (PDB ID: 1L8D) (Hopfner et al. 2002). The valine residue was identified by producing a uniformly deuterated, Ileδ1-[^13^CH_3_], Valγ-[^13^CH_3_/^12^CD_3_], Metε-[^13^CH_3_]-labeled sample (Lichtenecker et al. 2013). NOE-based assignments were extended by generating point mutants (L437I, L451I, L459I, and L460I) and comparing missing and/or shifted peaks in the 2D ^13^C,^1^H methyl-TROSY HMQC spectra. Stereospecific assignments of Leuδ/Valγ methyl groups were achieved by producing a pro-R labeled sample of ZHk (Gans et al. 2010). ZHk assignments were transferred to segmentally-labeled Rad50 in the MR complex through the comparison of 2D correlation spectra. Briefly, peaks in the 2D ^13^C,^1^H methyl-TROSY HMQC spectrum of segmentally-labeled Rad50 in the MR complex were assigned to the resonances in the isolated ZHk spectrum that were closest to their chemical shifts. Segmentally-labeled MR peaks with more than one plausible assignment from the ZHk spectrum were left unassigned.

^1^H R_2_ rates were determined by fitting peak intensities at relaxation delay times of 0.7, 12, 24, 36, 48, 60, 90, and 120 ms for ZHk and 0.7, 6, 12, 24, 36, 48, and 60 ms for MR to monoexponential decay functions using in-house python scripts that make use of the *nmrglue* library (Helmus and Jaroniec 2013). Errors in the peak intensities were taken from the random noise in the spectra, and the resulting errors in the fitted relaxation rates were taken from the covariance of the fit.

## Results and discussion

Using a combined analysis of AFM data and computational approaches, flexible regions were identified within the coiled-coil regions of human RAD50 (van Noort et al. 2003). We used NCOILS Version 1.0 (Lupas et al. 1991) to predict areas of flexibility within the *Pf* Rad50 coiled-coils near the Zn Hook motif (Fig. 2) and chose these sites as boundaries between segments 1 and 2 and segments 2 and 3 for segmental labeling. Thus, the ligation sites are in conserved regions of flexibility and should not disrupt the structure of the coiled-coils. The N-terminus of Rad50 (Ncc) is insoluble if not co-expressed with the C-terminus of Rad50 (ccC). Therefore, unlabeled segments 1 and 3 must be co-expressed, and the two ligation reactions to insert the side-chain methyl group-labeled ZHk segment (segment 2) occur on this folded protein complex. As a result of the co-expression, orthogonal ligation methods were needed as the two ends would be in proximity in the Ncc + (6HisTEV)ccC complex. Initially, we designed a ligation scheme employing IPL to ligate segments 1 and 2 and SrtA ligation for segments 2 and 3. However, successful IPL was unattainable likely due to the high degree of flexibility between segments 1 and 2. We then chose to use OaAEP1 as a second ligation enzyme. In this scheme, we kept the ZHk (segment 2) in the pTWIN vector and used its pH inducible *Ssp* DNAb-derived Intein to cleave off the chitin binding domain and leave an N-terminal Gly-Leu sequence as an OaAEP1 substrate. Segment 1 (Ncc) was also kept in the pTWIN vector, but a stop codon was inserted before the Intein.

### Optimization of OaAEP1 ligation reaction

**Fig. 3:**
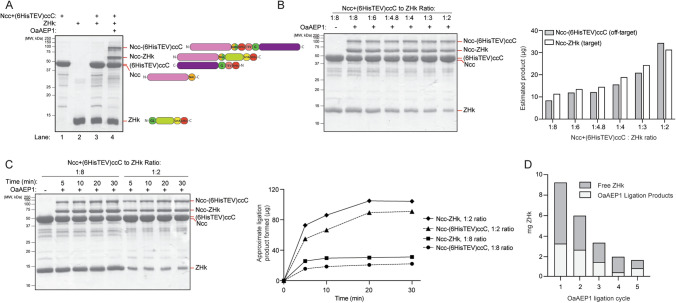
Analysis of OaAEP1 ligation reactions. **A** 15% SDS-PAGE analysis of OaAEP1-catalyzed ligation between Ncc and ZHk. Lanes 1 and 2: Ncc + (6HisTEV)ccC and ZHk reactants loaded separately. Lane 3: mixture of the two reactants (30 µM Ncc + (6HisTEV)ccC and 100 µM ZHk) before the addition of 0.4 µM OaAEP1. Lane 4: 20 min at room temperature after the addition of OaAEP1. The quantity of loaded samples was kept constant at 40 nmol of Ncc + (6HisTEV)ccC and the ratio-equivalent nmol for ZHk. On the right are cartoons of the Rad50 peptide in each band indicated on the gel. **B** OaAEP1 ligation yields of Ncc-ZHk (target) and Ncc-(6HisTEV)ccC (off-target, “semi-cyclized”) products at various Ncc + (6HisTEV)ccC:ZHk ratios. Graph on right was made from ImageQuant analysis of bands on gel on left. **C** OaAEP1 ligation yields of target and “semi-cyclized” products at various time points for 1:8 and 1:2 Ncc + (6HisTEV)ccC:ZHk ratios. Graph on right was made from ImageQuant analysis of bands on gel on left. **D** Approximate yields of reacted (white bar) and unreacted (gray bar) ZHk as a function of recycling cycle number as calculated by the UV absorbance after Q column purification of the OaAEP1 ligation reaction (Fig. 1B, Steps 1 and 2). The ZHk represented in each gray bar was used as the substrate in the subsequent ligation cycle

Prior to large-scale generation of segmentally labeled full-length Rad50 for NMR samples, each ligation step was carefully optimized with the goal of maximum yield of ligated product. The products of the OaAEP1 ligation and subsequent purification steps were analyzed by SDS-PAGE. Consistent with previous literature, we found that the C247A mutant of OaAEP1 led to faster generation of products compared to the wild type enzyme (Yang et al. 2017). In Step 1, the OaAEP1 ligation yielded multiple products, as indicated by the emergence of higher molecular weight species on the SDS-PAGE gel (Fig. 3A). The sizes of the two bands above the unreacted Ncc and (6HisTEV)ccC species corresponded to those for Ncc ligated to ZHk (Ncc-ZHk; 60.7 kDa) and an off-target semi-cyclized product consisting of Ncc and (6HisTEV)ccC joined at their C- and N-terminal ends, respectively (Ncc-(6HisTEV)ccC; 94 kDa). This off-target ligation event likely occurs because of the catalytic efficiency of OaAEP1 and the proximity of the ends due to the interaction of the coiled-coil domains in segments 1 and 3. Our assumption that the band around 100 kDa formed after OaAEP1 ligation is the semi-cyclized product was supported by gel data which showed an increase in this product when the Gly on the N-terminus of ccC is exposed after TEV cleavage. Furthermore, the band formed around 50 kDa after TEV cleavage (Fig. 1B, Step 3) is consistent with the molecular weight of TEV-cleaved (and therefore, de-cyclized) Ncc-(6HisTEV) product and was removed during the final HisTrap HP purification step (Fig. 1B, Step 3).

The incorporation of methyl-labeled ZHk substrate in Step 1 was dependent on the relative concentration of Ncc + (6HisTEV)ccC and ZHk, as expected (Deng et al. 2020). Estimation of target and off-target product yields from gel band intensities suggested increasing product formation with larger Ncc + (6HisTEV)ccC:ZHk ratios (Fig. 3B). Regardless of substrate concentrations, the OaAEP1 reaction slowed considerably after 20 min (Fig. 3C). Despite the relation of OaAEP1 to asparaginyl endopeptidases and the presence of multiple Asn residues in Rad50, the gels showed no evidence of protein degradation after addition of OaAEP1 (Fig. 3) (Mikula et al. 2017). Nevertheless, we aimed for minimum reaction times to limit the formation of the semi-cyclized product. Although enzyme activity fluctuates between each prep, we have found that 0.3–0.5 µM is a suitable concentration of OaAEP1 for ligation reactions when the Ncc + (6HisTEV)ccC:ZHk ratio is less than 1:2.

After purification of the OaAEP1 ligation reaction on the nickel column (Fig. 1B, Step 2), there was no further product formation, suggesting successful elimination of OaAEP1. Quantification of the yield of the products after Steps 1 and 2 helped us to understand productive substrate incorporation (i.e., successful OaAEP1 ligation) and unproductive loss of labeled ZHk due to the various purification steps (i.e., HisTrap and Q columns). Because of the difficulty in determining the concentrations of the desired product within a solution of multiple species, relative yields were represented by quantification of free ZHk in the Q column flow through after Step 2. The analysis showed a steady recovery of unreacted free ZHk in the Q column flow through following successive ligation cycles, indicating a lower yield of the desired product after multiple OaAEP1 ligation reactions (Fig. 3D). Thus, we determined that the process of recycling unreacted ZHk can be repeated four or five times to optimize for ligation of the ILVM-labeled segment 2 to Ncc segment 1 (Figs. 1B and 3D).

### Optimization of SrtA ligation reaction

TEV protease processing proceeded immediately after Step 2 (i.e., Fig. 1B, Step 3) and finished within one to two days. Purification on the nickel column removed most of the unreacted substrates from the OaAEP1 ligation reaction. In Step 4 (Fig. 1B), the SrtA enzyme produces full-length Rad50 by replacing the “G-6His” tag on the C-terminus of ZHk with the exposed N-terminal Gly on ccC, which resulted from the aforementioned TEV cleavage. The SrtA ligation reaction results in a small peptide by-product, (GHHHHHH), which must be continually removed from the reaction to prevent SrtA from using it as a substrate and displacing the successfully ligated Rad50 segment 3 (Freiburger et al. 2015).

To optimize the SrtA reaction, we combined the procedures of other groups, including ligating at a higher temperature and continual elimination of the undesired small peptide product (Freiburger et al. 2015). The possibility of hydrolysis was also a reason to limit incubation time (Freiburger et al. 2015). Because the elimination of the small peptide product (GHHHHHH) as flow through in a centrifugal concentrator involved many transfer steps and loss of protein bound to the membrane, we decided that performing dialysis during the reaction incubation at 37 °C was the better way to achieve both goals of product elimination and limited hydrolysis.

**Fig. 4:**
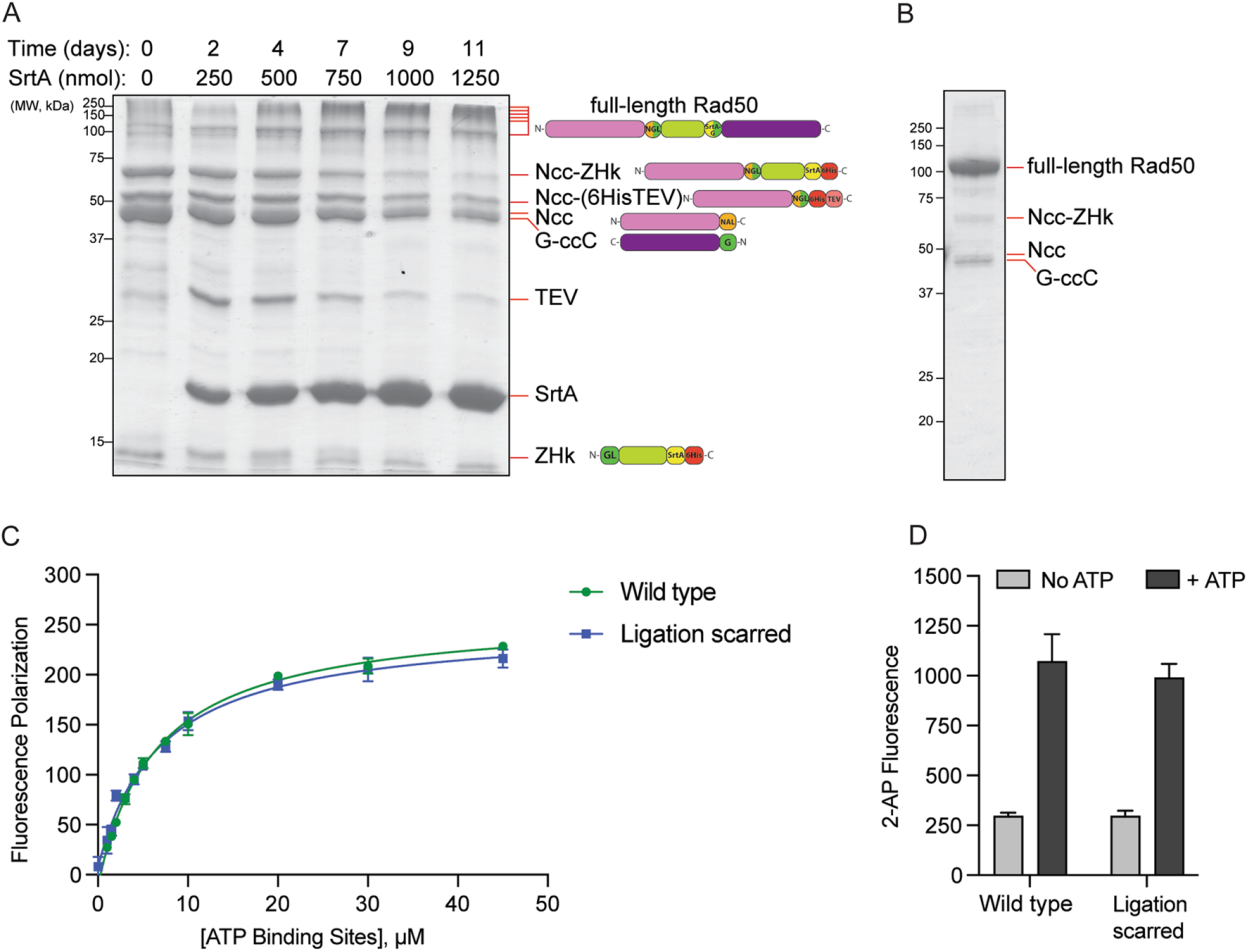
SrtA ligation reaction and purified ligated Rad50. 15% SDS-PAGE showing the progression of the SrtA ligation reaction with TEV-cleaved Ncc-ZHk + ccC over 11 days. Additional SrtA is added to the on-going reaction at 2–3 day intervals. On the right are cartoons of the Rad50 peptide in each band indicated on the gel. Full-length Rad50 oligomerizes and runs as a smear at the top of the gel in addition to its expected ~ 104 kDa band. **B** 12% SDS-PAGE of S200-purified segmentally ligated Rad50. **C** Polarization of fluorescently labeled ATP as a function of the concentration of MR ATP binding sites in wild type and ligation-scarred MR complexes. Data points are presented as the average and standard deviation of three replicates. Solid lines represent fits to a standard binding equation. **D** Fluorescence of 2-aminopurine (2-AP) nucleotide analog in the presence of wild type and ligation-scarred MR complexes. Gray and black bars represent nuclease activity assays performed in the absence and presence of 2 mM ATP/5 mM MgCl_2_, respectively. Bars represent the average and standard deviation of three replicates

As suggested by other groups, the SrtA ligation process takes several days and requires large quantities of enzyme (Jeong et al. 2017). Qualitatively, we found that the formation of ligation product in our system plateaued after 9 days (Fig. 4A). We observe that full-length Rad50 runs both as a distinct band at ~ 100 kDa on the SDS-PAGE gel and as a smear of higher molecular weight bands above that. Rad50 is known to oligomerize (Zabolotnaya et al. 2020; Kissling et al. 2022), and we hypothesize that the high MW bands result from the oligomers. Final purification with a nickel affinity column left an intermediate product around 45 kDa, residual ZHk, and SrtA in the mixture; however, these contaminants are removed following size exclusion chromatography purification (Fig. 4B). For 1429 nmol starting sample of ZHk, we have generated 340 nmol of ligated Rad50, a roughly 24% yield, and 309 nmol of leftover ZHk. Considering we obtain about 2430 nmol ZHk per liter of deuterated media, this ligation scheme yields about 583 nmol (61 mg) of segmentally labeled full-length Rad50 per liter of D_2_O.

### Ligation does not affect MR activity

Since our ligation scheme leaves six mutations in the Rad50 coiled-coil regions (TPLL → PLTG for SrtA and LGD → NGL for OaAEP1, Fig. 2), Rad50 and Mre11 activities were assayed to test for any effect that these mutations might have on MR complex function. An identical K_D_ for ATP binding was observed for the ligation-scarred Rad50 MR complex as compared to MR complex containing wild type Rad50 (Fig. 4C). This was not surprising as ATP binds to the globular NBD domains of Rad50 and likely does not involve the coiled-coils. When Mre11 nuclease activity was tested, MR complex made with either wild type or ligation-scarred Rad50 cleaved a dsDNA substrate with equal efficiency (Fig. 4D). Note, in this fluorescence-based nuclease activity assay, where cleavage of a modified nucleotide at the eleventh position generates the observed signal, efficient cleavage by Mre11 requires full-length Rad50 and hydrolysis of ATP by Rad50 to “processively” cleave the dsDNA (Herdendorf et al. 2011; Deshpande et al. 2014; Boswell et al. 2020). Thus, these activity assays demonstrated that the mutations generated for the ligation reactions had no effect on MR activity.

### Ligati﻿﻿on validation via NMR spectroscopy

**Fig. 5:**
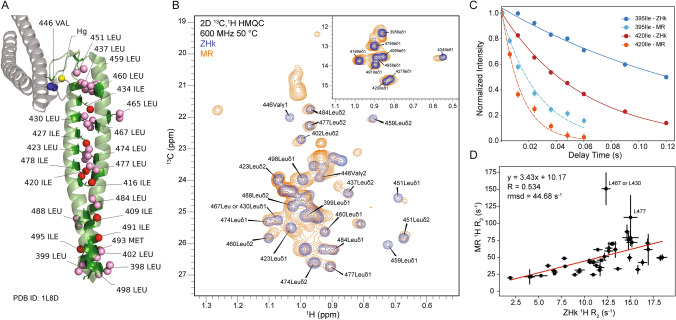
NMR on isolated and ligated side-chain methyl group-labeled ZHk domain. **A** Crystal structure of *Pf* Rad50 Zn Hook (PDB ID: 1L8D) with side-chain methyl group residues labeled (Hopfner et al. 2002). **B** 2D ^13^C,^1^H correlation spectra of labeled leucine/valines and isoleucines (top right inset) for isolated ZHk (blue contours) and segmentally-labeled (orange contours) *Pf* Rad50 within the MR complex. **C** Methyl ^1^H R_2_ relaxation decay curves for 395IleCδ1 and 420IleCδ1 methyl groups in isolated ZHk (darker points and solid curves; 6.0 ± 0.3 and 16.9 ± 0.3 s^−1^, respectively) and segmentally-labeled Rad50 within the MR complex (lighter points and dashed curves; 37.7 ± 3.1 and 70.9 ± 6.8 s^−1^, respectively). **D** Correlation of ZHk side-chain methyl group ^1^H R_2_ relaxation rates for ZHk residues in MR complex *versus* isolated ZHk. The red line represents the line of best-fit to the data points, which is given along with Pearson’s correlation coefficient (R) and root mean square deviation (rmsd)

To confirm that the ILVM-labeled ZHk was successfully ligated into place within full-length Rad50, the 2D ^13^C,^1^H methyl-TROSY HMQC correlation spectra of isolated ZHk and segmentally-labeled Rad50 in complex with unlabeled Mre11 (MR) were compared. Note, Rad50 on its own (i.e., without Mre11) tends to oligomerize at NMR concentrations, so we were not able to directly compare the isolated ZHk to ligated ZHk. Because the globular domains of the MR complex are > 600 Å away from the ZHk, there should not be an issue in comparing the NMR data of the isolated ZHk to the MR complex. We observed chemical shift perturbations in the HMQC spectra of the ZHk in the MR complex for the methyl groups near the ligation sites as well as for several residues near the Zn^2+^ binding region (Figs. 5A and 5B). Some residues, like L437, L423, and I434, developed elongated or adjacent twin peaks in the MR complex. Other residues located in the ZHk, like L451 and L459, show slight chemical shift perturbations and marked reductions in intensities. In the isoleucine region, the largest chemical shift changes corresponded to the residue closest to the ligation sites (I395) and the one closest to the zinc ion (I434). The movement of peaks for methyl groups at the ligation sites is consistent with a change in structure due to ligation of the isolated ZHk domain into the full-length Rad50 protein. Other chemical shift perturbations distal to the site of ligation could hint at allostery within the ZHk domain.

To further confirm the presence of ligated ZHk in our segmentally labeled MR complex, we also measured methyl ^1^H R_2_ data, which are sensitive to global tumbling of the molecule and local motions of the probe. Side-chain methyl group ^1^H R_2_ relaxation data showed an average increase in methyl ^1^H relaxation rates by a factor of 3.43 in the ligated Rad50 in the MR complex compared to isolated ZHk (Figs. 5C and 5D). This suggests a decrease in the tumbling of the domain upon incorporation into MR. We do not expect this increase to be 100% proportional to the mass difference between isolated ZHk and full-length Rad50 (Table 1) because of its fibrous structure and the known flexibility of the coiled-coils (van Noort et al. 2003). Nonetheless, the NMR data confirm that the dual enzymatic ligation method was successful in the three segment ligation of *Pf* Rad50.

## Summary

Herein, we described a method to localize isotopic labels to the internal ZHk domain of Rad50 by ligating three segments of the protein together with OaAEP1 and SrtA. To the best of our knowledge, this is the first use of two orthogonal enzymatic ligation reactions to join three parts of a full-length protein. By strategically positioning the ligation site in regions of conserved flexibility within the Rad50 coiled-coils, we minimized any effects of ligation scars on MR activities. Analysis of methyl-based NMR data revealed the expected chemical shift perturbations to side-chain methyl groups near the sites of ligation and the overall average increase in R_2_ relaxation rates that would accompany the incorporation of the methyl group probes into a larger protein. Theoretically, this method could be extended to ligate four segments, with minimal additional purification steps, in which two pairs are ligated separately with one of the enzymes and the full-length molecule constructed with the other enzyme.
